# Sensitive Detection of Multi-Point Temperature Based on FMCW Interferometry and DSP Algorithm

**DOI:** 10.3390/nano15201545

**Published:** 2025-10-10

**Authors:** Chengyu Mo, Yuqiang Yang, Xiaoguang Mu, Fujiang Li, Yuting Li

**Affiliations:** 1 Research Center of Guangdong Smart Oceans Sensor Networks and Equipment Engineering, Guangdong Ocean University, Zhanjiang 524088, China; chengyumo11@163.com; 2 Guangdong Provincial Key Laboratory of Intelligent Equipment for South China Sea Marine Ranching, Guangdong Ocean University, Zhanjiang 524088, China; liyuting5892022@163.com; 3 Information System Integration Center of China Mobile Group Shandong Co., Ltd., Jinan 250101, China; lifujiang@sd.chinamobile.com

**Keywords:** optical fiber sensing technology, frequency modulation continuous wave, modulation function, virtual Vernier effect, interference measurement

## Abstract

This paper presents a high-sensitivity multi-point seawater temperature detection system based on the virtual Vernier effect, achieved through multiplexed Fabry–Perot (FP) cavities combined with optical frequency-modulated continuous wave (FMCW) interferometry. To address the nonlinear frequency scanning issue inherent in FMCW systems, this paper implemented a software compensation method. This approach enables accurate positioning of multiple FP sub-sensors and effective demodulation of the sensing interference spectrum (SIS) for each FP interferometer (FPI). Through digital signal processing (DSP) algorithms and spectral demodulation, each sub-FP sensor generates an artificial reference spectrum (ARS). The virtual Vernier effect is then achieved by means of a computational process that combines the SIS intensity with the corresponding ARS intensity. This eliminates the need for physical reference arrays with carefully detuned spatial frequencies, as is required in traditional Vernier effect implementations. The sensitivity amplification can be dynamically adjusted with the modulation function parameters. Experimental results demonstrate that an optical fiber link of 82.3 m was achieved with a high spatial resolution of 23.9 μm. Within the temperature range of ${30\;}^{\circ }C$ to ${70\;}^{\circ }C$, the temperature sensitivities of the three enhanced EIS reached −275.56 $\;\mathrm{pm}{/}^{\circ }C$, −269.78 $\;\mathrm{pm}{/}^{\circ }C$, and −280.67 $\;\mathrm{pm}{/}^{\circ }C$, respectively, representing amplification factors of 3.32, 4.93, and 6.13 compared to a single SIS. The presented approach not only enables effective multiplexing and spatial localization of multiple fiber sensors but also successfully amplifies weak signal detection. This breakthrough provides crucial technical support for implementing quasi-distributed optical sensitization sensing in marine environments, opening new possibilities for high-precision oceanographic monitoring.

## 1. Introduction

Accurate monitoring of ocean temperature is of critical importance for understanding climate change, studying marine ecosystems, and forecasting extreme weather events [1]. It serves as a fundamental parameter in oceanographic research, fishery management, and the development of global climate models. Conventional techniques for measuring seawater temperature, such as ship-based water sampling or expendable bathythermographs (XBTs), although widely used, are limited by low spatial resolution, high operational costs, and the difficulty in achieving long-term continuous vertical profiling [2]. While satellite remote sensing can provide large-scale sea surface temperature data, its penetration depth is limited, and it is susceptible to interference from clouds and atmospheric conditions, making it inadequate for high-precision vertical temperature measurements [3]. In recent years, the utilization of fiber-optic sensing technology has emerged as a promising solution for ocean temperature monitoring. This technology boasts a number of inherent advantages, including immunity to electromagnetic disturbance, corrosion resistance, compact size, light weight, and long-distance transmission capability [4,5,6,7,8,9]. Among various configurations, point-type sensors based on Fabry–Pérot interferometers (FPIs) or fiber Bragg gratings (FBGs) have demonstrated excellent performance [10,11,12,13]. However, these sensors are inherently discrete by nature, providing temperature measurements only at specific points. Achieving large-scale, high-spatial-resolution distributed sensing would require the deployment of a large number of sensor nodes, leading to significantly increased system complexity and cost, which poses considerable challenges for practical applications.

In this context, the introduction of frequency-modulated continuous wave (FMCW) interferometry, a technique adapted from radar systems, has effectively addressed these limitations. This approach is known in the optical domain as optical frequency domain reflectometry (OFDR). It leverages the backscattered Rayleigh signal within an optical fiber to not only locate physical events at any position along the fiber link but also enable distributed sensing of environmental temperature and structural strain, as demonstrated in multiple studies [14,15,16,17,18,19,20,21]. While OFDR technology enables long-range and large-area sensing, its signal demodulation relies on the intensity of backscattered light rather than interferometric spectral information due to fundamental sensing principles. This intensity-based approach results in relatively low measurement accuracy, as the optical signal is susceptible to fluctuations caused by environmental disturbances. In contrast, the integration of point-type sensors with FMCW technology forms a quasi-distributed sensing architecture that effectively combines the strengths of both techniques. This hybrid approach retains the high spatial resolution and precise localization capabilities of FMCW, while also incorporating the high responsivity and phase-based demodulation principles of point sensors, which significantly enhances overall sensing accuracy. For instance, Sun et al. devised a high-precision distributed-temperature-sensing system by leveraging the complex spectral characteristics of phase-shifted FBGs (PSFBGs) in conjunction with OFDR technology. The system attained a noteworthy localization precision of 20.5μm through multiplexing an array of 1974 ultra-weak PSFBGs, with each individual PSFBG exhibiting a temperature sensitivity of approximately 10.4 pm/°C [22]. In another study, Zhu et al. proposed and experimentally validated a novel multiplexing approach for Fabry–Pérot (FP) sensors within a multi-branch configuration using FMCW interferometry. Through hardware-based compensation, nonlinear errors in the system were effectively mitigated, enabling temperature sensing with a maximum sensitivity of 0.185 nm/°C across a range of 20–80 °C [23,24]. Furthermore, Feng et al. employed a software compensation strategy to suppress nonlinear tuning effects in an OFDR system. Over a sensing length of 13.6 m, they successfully multiplexed six FP sensors to realize simultaneous temperature-compensated humidity measurements, with corresponding sensitivities of 32.77 pm/°C and −157 pm/%RH, respectively [25]. Although FMCW-FPI quasi-distributed sensing systems have significantly extended the measurement range, their relatively low detection sensitivity remains a major constraint for broader applications.

In order to enhance the detection sensitivity of optical fiber sensors, the optical Vernier effect has emerged as a technical approach that has been adopted on a wide scale. This effect is achieved by integrating two interferometers with closely matched free spectral ranges (FSRs) in a cascaded or parallel configuration, functioning as the sensing and reference units, respectively. When the interference spectra of these two units overlap, a composite spectrum featuring an envelope is generated. The envelope’s wavelength shift substantially exceeds that of a single interferometer upon external parameter variation for significant signal amplification. This mechanism endows Vernier-effect-based composite interferometric structures with superior sensitivity, making them highly suitable for high-precision detection of minute changes. Shao et al. developed a cascaded structure consisting of two Fabry–Pérot interferometers (FPIs), utilizing the resulting Vernier effect to enhance the temperature sensitivity to 9.1 times that of a single FPI [26]. Chen et al. developed a temperature sensor utilizing two cascaded Mach–Zehnder interferometers (MZIs), employing Fourier transform analysis of the superimposed spectra to demodulate the Vernier effect and extract the beat frequency, achieving a measured temperature sensitivity of 397.36 pm/°C [27]. Similarly, Liu et al. employed a cascaded two-FPI structure to generate the Vernier effect, reporting a temperature sensitivity enhancement of 10.77 times relative to a single interferometer [28]. Nevertheless, such sensors still face several technical challenges. The primary issues include the difficulty in matching the optical path difference (OPD) between the sensing interferometer (SIM) and the reference interferometer (RIM), as well as the RIM’s vulnerability to external environmental disturbances in pragmatic applications. Moreover, achieving high sensitivity across a quasi-distributed sensor array necessitates pairing each sensing unit with a corresponding physical RIM designed with a specific OPD. This demands precise hardware design to ensure performance, which considerably increases the complexity of implementation and the potential for measurement errors.

In order to address the aforementioned challenges, this article proposes a multi-point sensing technique based on FMCW interferometry and the virtual Vernier effect. The former is employed for sensor localization and interferometric spectrum demodulation within the sensing array, while the latter is utilized for the enhancement of the sensitivity of each sensor. This approach employs only a predefined modulation function applied to the sensing interference spectrum (SIS) of each sensor to produce an artificial reference spectrum (ARS). Multiplying the SIS with the ARS yields an envelope interference spectrum (EIS) exhibiting the virtual Vernier effect. Similar to the conventional Vernier effect, the virtual EIS demonstrates an amplified wavelength shift relative to the sensor’s original SIS under increasing temperature. To validate this technique, an open-cavity FPI array was fabricated and deployed in a multi-point seawater temperature sensing experiment. Experimental results demonstrate that the proposed system, incorporating digital signal processing (DSP) algorithms, achieved a fiber link monitoring length of 82.3 m with a high spatial resolution of 23.9μm. Furthermore, across the temperature range of 30–70 °C, a sensitivity amplification factor of 6.13 was attained compared to a single sensor. In contrast to conventional Vernier-effect-based approaches, the proposed method offers several distinct advantages: a simpler structure, reduced footprint, and highly flexible tunability of sensitivity. Moreover, instead of physically fabricating reference interferometers, the system generates an array of virtual reference spectra algorithmically based on the demodulated interference spectra of the FPIs, thereby effectively emulating the Vernier effect in software. This approach significantly reduces system complexity and cost.

## 2. Multi-Point Location and Sensitization Principle

### 2.1. Location of Sensors

The optical FMCW interferometric system incorporates an MZI consisting of reference and sensing arms. A frequency-swept beam from a tunable laser source (TLS) is divided by an optical coupler into reference and sensing beams that propagate through their respective arms. Assuming a chirp rate of γ Hz/s for the incident light, the electric fields of the reference light and the sensing light can be represented as follows [29]:

(1) $$E_{r}\left(t\right)=\sqrt{a}E_{0}e^{j(2\pi f_{0}t+\pi \gamma t^{2}+\phi \left(t\right))}$$

(2) $$E_{s}\left(t\right)=\sqrt{R\left(\tau_{z}\right)\left(1-a\right)}E_{0}e^{j(2\pi f_{0}\left(t-\tau_{z}\right)+\pi \gamma {\left(t-\tau_{z}\right)}^{2}+\phi \left(t-\tau_{z}\right))}$$

where *a* denotes the splitting ratio of the coupler, $E_{0}$ represents the initial electric field amplitude emitted from the TLS, and $f_{0}$ is the starting frequency of the frequency-swept light. The term ϕ(t) corresponds to the phase noise, which arises from carrier fluctuations and temperature drift during active tuning of the laser source. The spectral broadening of the beat signal is primarily accounted for by this inherent chirping error. $R(\tau_{z})$ denotes the reflectivity at point *z* along the sensing arm, where $\tau_{z}=2nz/c$ represents the time delay difference in the optical path between point z and the reference arm. The term *n* denotes the equivalent refractive index (RI) at the reflection point, and *c* represents the speed of light in a vacuum. Considering that the reflections in the sensing arm originate from a cascaded FP sensor array, the interference intensity formed by the reference light and the multi-beam reflected light after the DC component is removed can be described as follows:

(3) $$\begin{array}{cc} I(t) & =\left[E_{r}\left(t\right)+E_{\mathrm{sm}}\left(t\right)\right]{\left[E_{r}\left(t\right)+E_{\mathrm{sm}}\left(t\right)\right]}^{*}\\ \; & =E_{all}\sum_{1}^{m}\left[R\left(\tau_{zm}\right)\cos\left(2\pi f_{0}\tau_{zm}+2\pi \gamma \tau_{zm}t-\pi \gamma \tau_{zm}^{2}+\phi \left(t\right)-\phi \left(t-\tau_{zm}\right)\right)\right] \end{array}$$

where $R(\tau_{zm})$ and $\tau_{zm}$ represent the reflection coefficient and the temporal delay of the *m*-th reflection position, respectively. $E_{all}=2E_{0}^{2}\sqrt{a(1-a)}$ is the total light intensity amplitude. As indicated in Equation (3), the intermediate frequency (IF) signal commonly referred to as the beat frequency is generated through the mixing of the reference light and the sensing light. This frequency, given by $f_{b}=\gamma \tau_{z}=\left(2nz\gamma \right)/c$, shows a linear correlation with the position of the backscattered signal along the sensing fiber. Therefore, the application of a Fourier transform to the overall interference signal can converts the time domain to the distance domain, enabling precise localization of all reflection points. Subsequently, the sensing spectrum of the FPI is obtained by filtering and extracting the spectral information from the reflected signal.

### 2.2. Modulated Vernier Sensitization Principle

When the frequency-swept light emitted from the TLS encounters the two highly reflective surfaces of the FPI micro-cavity, a two-beam interference phenomenon occurs. Based on the two-beam interference principle, the initial interference spectrum of an FP sensor can be described by [30]

(4) $$I_{0}\left(\lambda \right)=A+B\cos\left(\frac{4\pi n_{0}L}{\lambda }\right)$$

where *A* and *B* correspond to the effective reflected light intensity from the two end faces of the FPI micro-cavity, $n_{0}$ is the initial effective RI of the FPI prior to sensing, *L* represents the cavity length, and λ is the central wavelength of the incident light. Given that the thermal expansion coefficient of the quartz tube is $\beta =5.5\times 10^{-7}{/}^{\circ }C$, while the coefficient of the thermo-optical effect in seawater is α=$-1\times 10^{-4}{/}^{\circ }C$, the change in refractive index overwhelmingly dominates the spectral response. Therefore, the wavelength shifts $\Delta \lambda_{m}$ of the FPI are given by

(5) $$\Delta \lambda_{m}=\lambda_{m}\left(\frac{\Delta L}{L}+\frac{\Delta n}{n_{s}}\right)$$

where $\lambda_{m}$ denotes the resonant wavelength corresponding to the *m*-th interference peak, ΔL denotes the change in cavity length, and Δn denotes the variation in the RI, with the actual sensing RI given by $n_{s}=n_{0}+\Delta n$. When the RI of the medium in the FP cavity varies due to environmental changes, it will cause a shift in the sensing spectrum. The measurement of this shift enables the calculation of the corresponding change in the target physical parameter. The temperature sensitivity of the FPI is given by

(6) $$S_{T}=\lambda_{m}\left(\frac{1}{L}\frac{dL}{dT}+\frac{1}{n}\frac{dn}{dT}\right)=\lambda_{m}\left(\alpha +\frac{1}{n}\beta \right)$$

Here, the proposed method synthesizes a virtual interference spectrum based on a DSP algorithm and modulates it with the sensing interference spectrum to generate an envelope spectrum, thereby amplifying the sensor’s sensitivity. It is worth emphasizing that this approach employs a virtual Vernier technique, eliminating the need for a physical reference interferometer as required in conventional implementations. Specifically, $I_{0}\left(\lambda \right)$ is modulated by the following modulation function [31]:

(7) $$I_{m}\left(\lambda \right)=\cos\left(\frac{4\pi }{\lambda }L_{m}\right)$$

where $L_{m}$ represents the length of the modulation. The modulated output, obtained by multiplying the modulation function (7) as the local oscillator signal with Equation (4), can be expressed as follows:

(8) $$I_{0}\left(\lambda \right)\times I_{m}\left(\lambda \right)=A\cos\left(\frac{4\pi }{\lambda }L_{m}\right)+\frac{B}{2}\cos\left(\frac{4\pi }{\lambda }\left(n_{0}L-L_{m}\right)\right)+\frac{B}{2}\cos\left(\frac{4\pi }{\lambda }\left(n_{0}L+L_{m}\right)\right)$$

As indicated in Equation (8), the modulation result comprises three components: The first is the baseband oscillation term, also referred to as the intermediate-frequency signal, which reflects the interference oscillation instigated by the fixed modulation difference $L_{m}$. The second is the difference-frequency component, corresponding to the low-frequency signal in the spectrum. The third component is the sum-frequency term, representing the high-frequency signal in the spectrum. Among these, the second term is selected as the ARS:

(9) $$I_{r}\left(\lambda \right)=\cos\left(\frac{4\pi }{\lambda }\left(n_{0}L-L_{m}\right)\right)$$

Moreover, by assigning $L_{m}$ to an arbitrary value, the FSR of the ARS can be made similar yet not identical to that of the SIS. Multiplying the experimentally measured SIS by the numerically constructed ARS yields an EIS exhibiting the Vernier effect:

(10) $$\begin{array}{cc} I_{Env}\left(\lambda \right) & =I_{s}\left(\lambda \right)\times I_{r}\left(\lambda \right)=A\cos\left(\frac{4\pi }{\lambda }\left(n_{0}L-L_{m}\right)\right)+\\ \; & \;\frac{B}{2}\cos\left(\frac{4\pi }{\lambda }\left(n_{s}L-n_{0}L+L_{m}\right)\right)+\frac{B}{2}\cos\left(\frac{4\pi }{\lambda }\left(n_{s}L+n_{0}L-L_{m}\right)\right) \end{array}$$

As shown in Equation (10), the multiplication result likewise consists of multiple components, corresponding, respectively, to the intermediate-frequency, low-frequency, and high-frequency terms in the spectrum. Since the second term contains the beat frequency information of the signal and manifests as the envelope of the interference signal, it is regarded as the envelope interference spectrum. Based on Equations (4), (9) and (10), the following expression can be derived:

(11) $$\left\{\begin{array}{c} FSR_{FPI}=\frac{\lambda^{2}}{2n_{0}L}\\ FSR_{r}=\frac{\lambda^{2}}{2n_{0}\left(L-L_{m}\right)}\\ FSR_{Env}=\frac{FSR_{r}\;\cdot \;FSR_{FPI}}{\left|FSR_{r}-FSR_{FPI}\right|} \end{array}\right.$$

Based on Equation (11), the magnification factor *M* of the sensing spectrum after applying the Vernier technique can be derived as follows:

(12) $$M=\frac{FSR_{r}}{\left|FSR_{r}-FSR_{FPI}\right|}$$

By selecting different FSRs for the ARS, the magnification *M* can be flexibly adapted, thereby enabling controllable tuning of the sensing sensitivity. The enhanced sensitivity can thus be expressed as follows:

(13) $$S_{T_{-}Env}=M\;\cdot \;S_{T}=M\lambda_{m}\left(\alpha +\frac{1}{n}\beta \right)$$

Therefore, when the ambient temperature changes, the Vernier envelope peak exhibits a uniform wavelength shift in response to the variation in the measured parameter. This shift is *M* times greater than that of a single SIS, thereby achieving enhanced sensitivity to the target parameter. In summary, a marked improvement in the temperature sensitivity of the FPI is achieved through the Vernier amplification technology.

## 3. Experiments and Discussions

### 3.1. Fabrication of Sensing Probes

**Figure 1:**
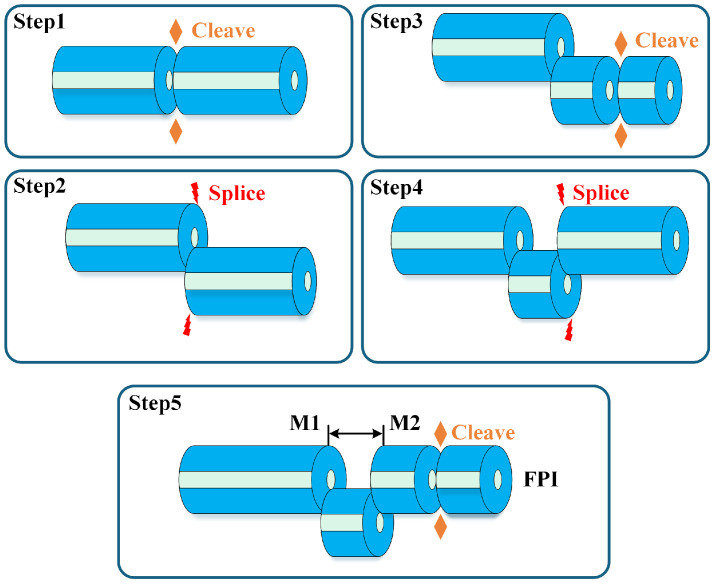
Fabrication process of the proposed fiber-optic sensing probe.

All temperature sensing probes used in this study for seawater applications were fabricated from standard single-mode fiber (SMF). To allow complete infiltration of seawater into the sensor, an open-cavity FPI structure was adopted. The detailed fabrication process is illustrated in Figure 1. In Step 1, the end face of the SMF was cleaved flat using a fiber cleaver. In Step 2, the fiber was fixed in a fusion splicer (Singalfire X) and subjected to a misaligned splice with an offset of 70μm, which fully exposed the fiber core to the external environment. In Step 3, the offset fiber was cleaved to a specific length to form the main body of the open cavity. In Step 4, another SMF with a flat-cleaved end face was similarly fusion-spliced with the same offset magnitude as in Step 2, but in the opposite direction. As illustrated in Step 5, after splicing, an open cavity was formed between the first reflective surface M1 and the second reflective surface M2, with the corresponding fiber cores of both reflective surfaces aligned on the same horizontal axis. Finally, the excess fiber was cleaved to an appropriate length to ensure that the spectral performance of the sensor remained unaffected. In this study, three FP sensors with cavity lengths of 207.49μm, 215.43μm, and 218.56μm, respectively, were fabricated for the demonstration of quasi-distributed multi-point temperature sensing based on FMCW. It is worth emphasizing that during the cleaving process in Step 1 and Step 3, the flatness of the fiber end face critically influences the spectral quality of the sensor. Therefore, the use of microscopy and real-time reflection spectrum monitoring is used to improve fabrication efficiency and consistency.

### 3.2. Experimental Setup and Operation Procedure

**Figure 2:**
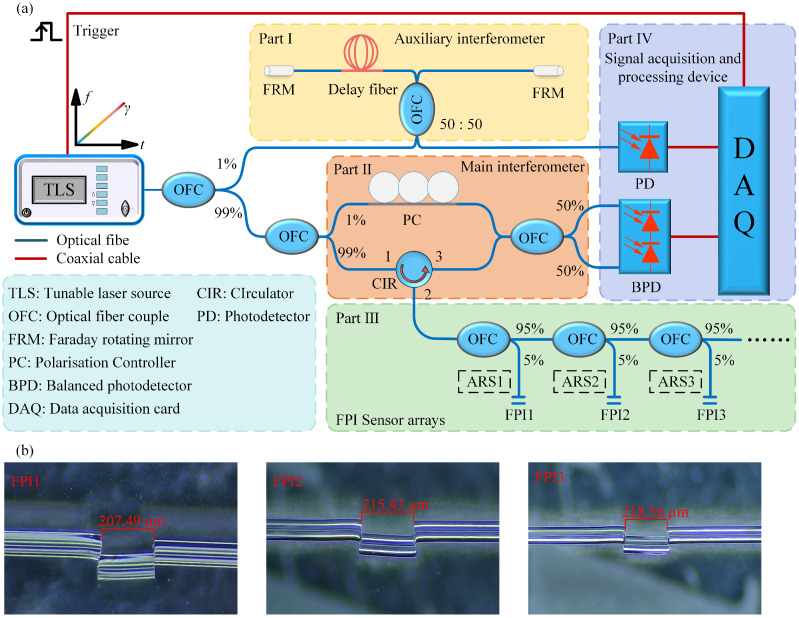
Experimental setup for multi-point temperature detection based on FMCW interferometry. (**a**) System construction flow chart; (**b**) micrograph of FP sensors.

The experimental setup for multi-point high-sensitivity temperature sensing is illustrated in Figure 2. A frequency-swept optical signal from a TLS is divided via an optical fiber coupler (OFC) into two separate paths. Part I functions as an auxiliary interferometer, acquiring real-time phase information from the TLS to compensate for nonlinear tuning effects. It consists of a Michelson interferometer with Faraday rotating mirrors (FRMs) providing a polarization-stable auxiliary beat signal. Notably, the software-based compensation method renders the actual sensing distance theoretically independent of the delay fiber length (30 m in this experiment). Part II forms the main interferometer, implemented via an MZI. To optimize the beat signal, the reference arm is equipped with a polarization controller. The sensing light is directed into Part III through an optical circulator, where an array of FPI sensors generates back-reflected signals varying with ambient temperature. The reflected signal interferes with the reference light to produce the main beat signal. Part IV comprises the signal acquisition unit: a photodetector converts optical signals to electrical ones, sampled by a DAQ card synchronized via a trigger signal from the TLS. To achieve high-sensitivity multi-point detection, a software-generated ARS is configured for each FPI and modulated with the SIS to emulate the Vernier effect, enabling enhanced sensitivity through monitoring the Vernier envelope.

This paper propose a DSP algorithm based on FMCW interferometry and multi-point Vernier-effect-based sensitivity enhancement. This algorithm not only achieves precise localization but also enables the multiplexing of FPIs and spectral sensitivity amplification. The specific steps of the DSP algorithm are as follows:

1. The raw signals from the main and additional interferometers are labeled as $U_{m1}\left(t\right)$ and $U_{a}\left(t\right)$, respectively. The instantaneous frequency *f* is obtained by applying the Hilbert transform and arctangent operation to $U_{a}\left(t\right)$.
2. The independent variable *t* in $U_{m1}\left(t\right)$ is replaced by the instantaneous frequency *f*, resulting in a new measurement signal $U_{m2}\left(f\right)$.
3. The nonlinear instantaneous frequency *f* is rearranged into a linearized version $f_{1}$. A one-dimensional linear interpolation and resampling of $U_{m2}\left(f\right)$ against $f_{1}$ yields the corrected signal $U_{m2}\left(t\right)$, thereby compensating for the nonlinear frequency sweep of the laser.
4. A Fast Fourier Transform (FFT) is applied to $U_{m2}\left(t\right)$ to obtain the corrected spatial domain information, enabling the localization of the FPI sensor array.
5. A bandpass filter is applied to the reflection peaks of the fiber micro-cavities, followed by an Inverse FFT (IFFT) to retrieve the wavelength-domain (time-domain) information. Under initial conditions, the envelope of the wavelength-domain signal is extracted to obtain the SIS of the fiber micro-cavity.
6. The SIS is modulated with the modulation function from Equation (7). An FFT is then applied to the modulated result, and the first peak is filtered and extracted to serve as the ARS for the FPI.
7. The software-generated ARS is multiplied with the experimentally acquired SIS. Subsequent spectral analysis is performed to select the envelope term, yielding the modulated Vernier envelope interference spectrum.
8. Finally, the same procedure is iterated for each FPI micro-cavity to achieve quasi-distributed and sensitivity-enhanced seawater temperature detection.

The experimental system utilized the following primary equipment: A tunable laser source (TLS; Agilent Technologies, 81640A; Santa Rosa, CA, USA) was employed, featuring a linewidth of 100 kHz, a scanning range of 1520–1570 nm, a sweep rate of 40 nm/s, and a step resolution of 10 pm. A photodetector (PD; KG-PR-200M-A-FC; Beijing Kangguan Shiji Photoelectric Technology Co., Ltd, Beijing, China) with a response bandwidth of 200 MHz and a detection wavelength range of 1100–1650 nm, was employed. A balanced photodetector (BPD; Thorlabs, PDB435C; Newton, NU, USA), covering a wavelength range from 800 to 1700 nm and possessing a bandwidth of 350 MHz, was also used. Data acquisition was performed using an Advantech acquisition card (DAQ; Advantech PCIE-1840; Advantech Co., Ltd., Taiwan, China), which supports 4 channels, 16-bit resolution, and a maximum sampling rate of 125 MHz per channel. Temperature control experiments were conducted using an temperature chamber (TC; HWS-50B; Mingtu Machinery Equipment Co., Ltd., Changge, China), providing a temperature range of $0–{70\;}^{\circ }C$ ($\pm {0.5\;}^{\circ }C$). The sensing fiber was a standard single-mode fiber (SMF; Corning SMF-28e; Corning, NY, USA), with a outer diameter of 125μm and a inner core diameter of 10μm.

### 3.3. Demodulation of Sensing Signal

**Figure 3:**
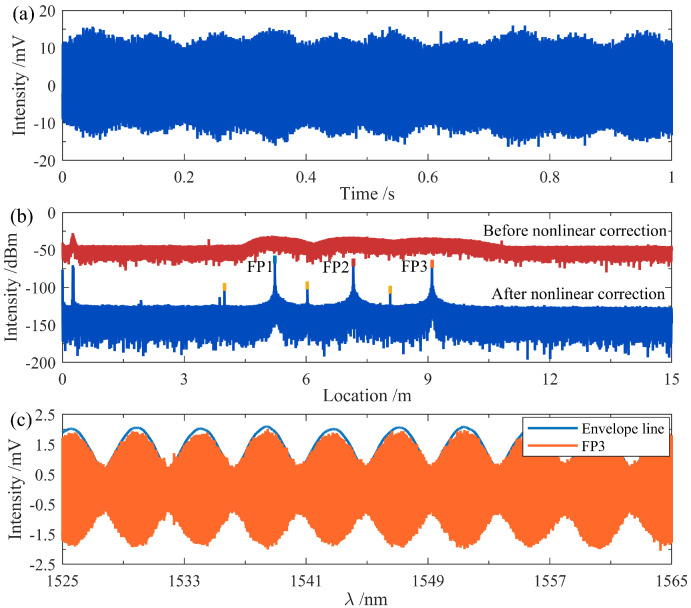
Signal demodulation process for the multi-point temperature sensing experiment. (**a**) Acquired raw signal; (**b**) comparison before and after nonlinear tuning compensation; and (**c**) demodulation of the FP3 interference spectrum.

The beat signal acquired under initial experimental conditions is shown in Figure 3a. The initial state was set at an ambient temperature of ${30\;}^{\circ }C$ and a seawater salinity of 35‰. The data acquisition card was configured with a sampling rate of 5 MHz, a sampling time of 1 s, and a total of $5\times 10^{6}$ sampling points. The distance-power profile obtained by applying FFT to the original beat signal is illustrated in Figure 3b. It can be observed that, due to the inherent nonlinear tuning issue of the system, significant spectral broadening occurred prior to compensation, making it impossible to accurately locate the sensors along the fiber link from the power spectrum (red curve). After compensation, distinct peaks emerged in the power spectrum (blue curve). The reflection peaks of the three sensors are marked in blue, red, and orange, located at optical distances of 5.2265 m, 7.1594 m, and 9.0966 m, respectively. The yellow marker indicates the fiber coupler connecting the three FP sensors. Subsequently, the reflection peak of the sensor of interest was filtered and extracted; here FP3 was selected. The original spectrum of FP3 obtained after Hanning window filtering is represented by the orange curve, as shown in Figure 3c, which results from the interference envelope of the two reflective surfaces of FP3 within the MZI structure. Finally, by extracting its outer envelope, the interference spectrum of FP3 was derived, as indicated by the blue envelope curve.

**Figure 4:**
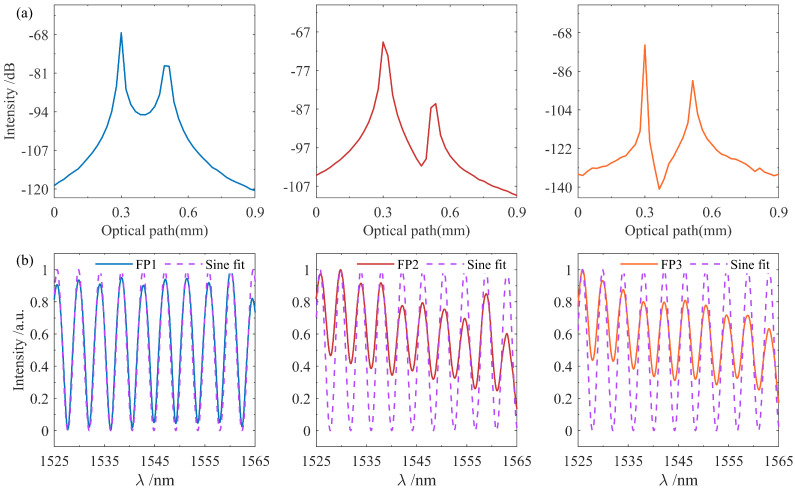
Detailed demodulation information of the FPIs. (**a**) Local enlarged images of three FPI reflection peaks; (**b**) normalized interference spectra of the three FPIs.

Figure 4 displays detailed demodulation information for the FPI sensor array. The reflection peaks from the two reflective surfaces of each sensor are clearly visible, as shown in Figure 4a, with the intensity of the first reflective surface being higher than that of the second, which is attributed to optical propagation loss within the sensing cavity. Meanwhile, the relative OPD between the two reflective surfaces corresponds to the FP sensor’s cavity length, enabling high-spatial-resolution localization. Based on the spatial resolution formula ΔZ=c/(2nΔF), where ΔF denotes the tuning speed of the TLS, the system’s achieved spatial resolution is 23.9μm. Figure 4b presents the normalized spectra of the three FPIs. Due to instability in the laser output spectrum, the original interference intensity of the FPI exhibited significant fluctuations. This issue was effectively addressed by applying sinusoidal fitting. Furthermore, the FSR of the FP sensors was determined through the measurement of the distance between two adjacent peaks or troughs in their SIS, yielding values of 4.34 nm, 4.18 nm, and 4.12 nm, respectively. These measured results exhibited close agreement with the theoretical predictions obtained from Equation (11). It is also worth noting that the actual sensing distance of the system was determined to be 82.3 m based on the distance formula described in reference [32].

### 3.4. Sensitization of Temperature Response

**Figure 5:**
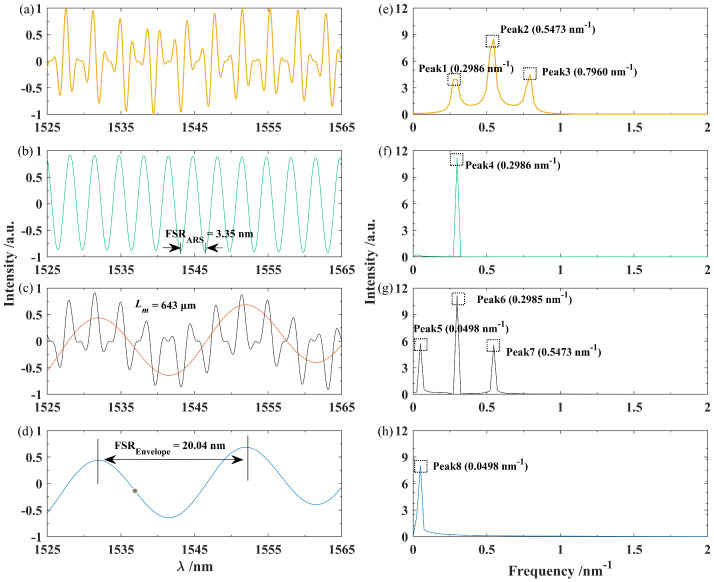
Construction of the ARS and sensitivity enhancement process of the interference spectrum for FP3. (**a**) Modulation result between the initial interference spectrum of FP3 and the modulation function; (**b**) ARS of FP3; (**c**) superimposed spectrum exhibiting the Vernier effect; (**d**) envelope interference spectrum extracted from (**c**); and (**e**–**h**) the FFT results for the signals in (**a**–**d**), respectively.

To achieve quasi-distributed multi-point sensitivity-enhanced sensing, the initially acquired FP interference spectrum was modulated for sensitivity amplification, with the modulation process of FP3’s SIS serving as an example. Figure 5a shows the spectrum resulting from multiplying the initial SIS of FP3 by the modulation function given in Equation (7). Here, the modulation length of the modulation function was set to 643μm, a value that can be arbitrarily assigned. Applying the FFT to the modulated spectrum yields the corresponding spatial spectrum, shown in Figure 5e, which exhibits three peaks corresponding to the low-frequency, intermediate-frequency, and high-frequency terms described in Equation (8). These peaks are located at Peak1 ($0.2986\;{\mathrm{nm}}^{-1}$), Peak2 ($0.5473\;{\mathrm{nm}}^{-1}$), and Peak3 ($0.7960\;{\mathrm{nm}}^{-1}$). As shown in Figure 5b, Peak1 was then extracted using a bandpass filter, and the spectrum obtained after applying the IFFT is presented as the ARS. Figure 5f displays the frequency profile corresponding to the ARS, where the value of Peak4 matches that of Peak1. Additionally, the FSR of the ARS can be derived as the reciprocal of the spatial frequency value. The calculated FSR was 3.348 nm, which is consistent with the experimentally measured value of 3.35 nm. By varying the modulation length $L_{m}$, reference spectra with diverse FSR values can be generated.

The constructed ARS was then multiplied with the interference spectrum of FP3 acquired under the sensing state, resulting in the superimposed spectrum exhibiting the Vernier effect, as shown in black in Figure 5c. A clear envelope can be observed in the superimposed spectrum, with the red curve representing its envelope trend line, i.e., the envelope interference spectrum used for sensitivity-enhanced sensing. Figure 5g shows the spatial frequency spectrum obtained by transforming the superimposed signal, which also shows three peaks corresponding to the low-frequency, intermediate-frequency, and high-frequency terms described in Equation (10). These peaks are located at Peak5 ($0.0498\;{\mathrm{nm}}^{-1}$), Peak6 ($0.2985\;{\mathrm{nm}}^{-1}$), and Peak7 ($0.5473\;{\mathrm{nm}}^{-1}$). Figure 5d displays the spectrum reconstructed after filtering and extracting Peak5, which corresponds to the envelope of the overlapped spectrum and exhibits an FSR of 20.04 nm. Figure 5h shows the FFT result of the envelope, where the value of Peak8 matches that of Peak5, confirming the feasibility of the proposed sensitivity enhancement method.

**Figure 6:**
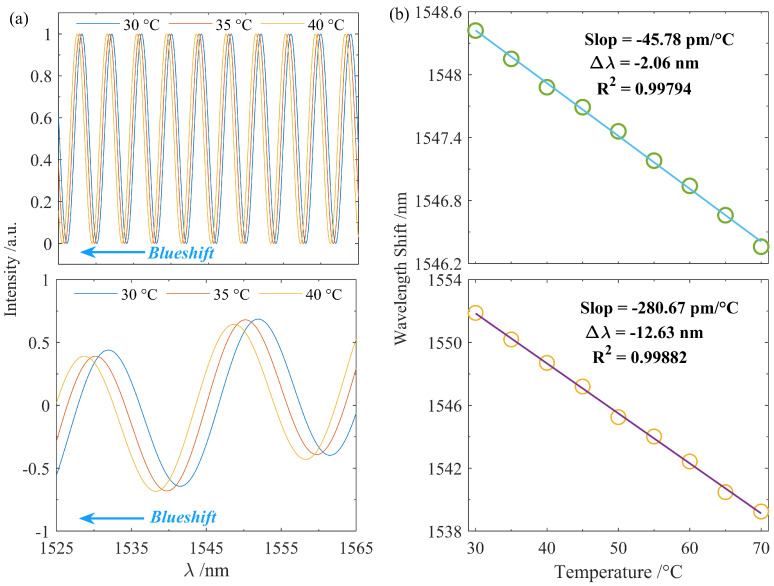
Temperature sensing characteristics of FP3. (**a**) Comparison of the sensing spectra before and after sensitivity enhancement; (**b**) linear fitting results of the sensor responses before and after sensitivity enhancement.

During the temperature sensing experiment, the three FP sensors were first placed in a container filled with standard seawater at a salinity of 35‰, ensuring complete immersion of the FPIs. While maintaining constant salinity, the entire sensing setup was placed inside a temperature chamber for controlled temperature variation. The temperature increased from ${30\;}^{\circ }C$ to ${70\;}^{\circ }C$ in steps of ${5\;}^{\circ }C$, with DSP-based measurements taken at each temperature point. Figure 6a illustrates the sensing behavior of the interference spectrum of FP3 within the temperature range of $30–{40\;}^{\circ }C$. It can be observed that both the SIS and EIS of FP3 exhibit a blue shift phenomenon before and after the application of Vernier-based sensitivity enhancement. This phenomenon occurs because the refractive index of seawater exhibits a negative correlation with temperature, leading to a reduction in the effective optical path length of the sensor and consequently a shift in the resonant wavelength toward shorter wavelengths. Furthermore, an markedly amplified wavelength shift is observed in the enhanced spectrum compared to the original interference signature. As shown in Figure 6b, the total wavelength shift of the original interference spectrum over the $30–{70\;}^{\circ }C$ temperature range was −2.06 nm, while that of the envelope interference spectrum reached −12.63 nm, corresponding to sensitivities of $-45.78\;\mathrm{pm}{/}^{\circ }C$ and $-280.67\;\mathrm{pm}{/}^{\circ }C$, respectively. Both sets of data exhibited linear fitting coefficients exceeding 99%. The achieved sensitivity amplification factor for the FP3 sensor was calculated to be 6.13.

**Figure 7:**
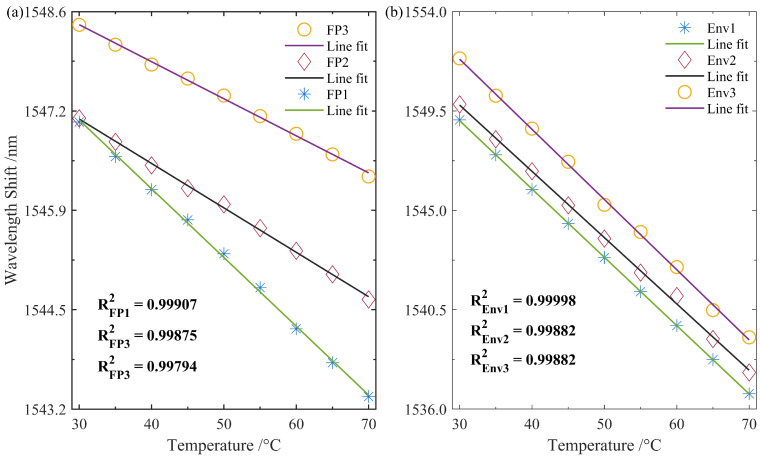
Temperature sensing response curves of the three sets of FP sensors. (**a**) Response curves of the original interference spectra of the three FPIs; (**b**) response curves of the envelope interference spectra of the three FPIs.

Similarly, the temperature sensing results of the remaining two sensors were demodulated. As shown in Figure 7a, prior to sensitivity enhancement, the linear fitting coefficients for the three FP sensors were 0.99907, 0.99875, and 0.99794, respectively. It can be observed that the sensitivity curves of the three sensors exhibit noticeable differences, which may be attributed to variations in their preset cavity lengths. Figure 7b presents the sensitivity curves of the envelope interference spectra for the three sensors after sensitivity amplification via the Vernier effect. Under the influence of the modulation function, the linear fitting coefficients of the sensing spectra improved significantly to 0.99998, 0.99882, and 0.99882, respectively. Furthermore, by adjusting the modulation length, the three FP sensors can be configured to exhibit similar sensitivity levels.

The detailed demodulation parameters of the three sets of sensors are summarized in Table 1. Since the same modulation length was applied to all FPIs during the sensitivity enhancement modulation process, their envelope interference spectra exhibit similar FSRs. Prior to enhancement, the FP1 sensor demonstrated relatively high sensitivity, which can likely be attributed to its shorter cavity length. A shorter FPI micro-cavity reduces the optical propagation path within the cavity, enabling a substantial increase in the effective number of interferences passes (i.e., photon round trips), thereby improving the practical sensitivity. After sensitivity enhancement, the three envelope spectra, which exhibit nearly identical FSRs, also demonstrate closely matched sensitivity. Through Vernier-based amplification, the temperature sensitivity of the FPIs was increased by factors of 3.32, 4.93, and 6.13, respectively. It is evident that the application of the modulated Vernier technique to distributed optical fiber sensing has significantly enhanced the sensing sensitivity of the FPIs.

The actual ambient temperature can be determined by solving the equations ΔT=Δλ/k and $T=T_{0}+\Delta T$, which link the wavelength shift of the interference spectrum to the temperature variation. Here, ΔT represents the temperature change, Δλ corresponds to the wavelength shift in the sensor’s spectrum, *k* is the sensitivity coefficient of the sensor, *T* refers to the actual temperature, and $T_{0}$ indicates the calibrated reference temperature.

### 3.5. Stability of System

**Figure 8:**
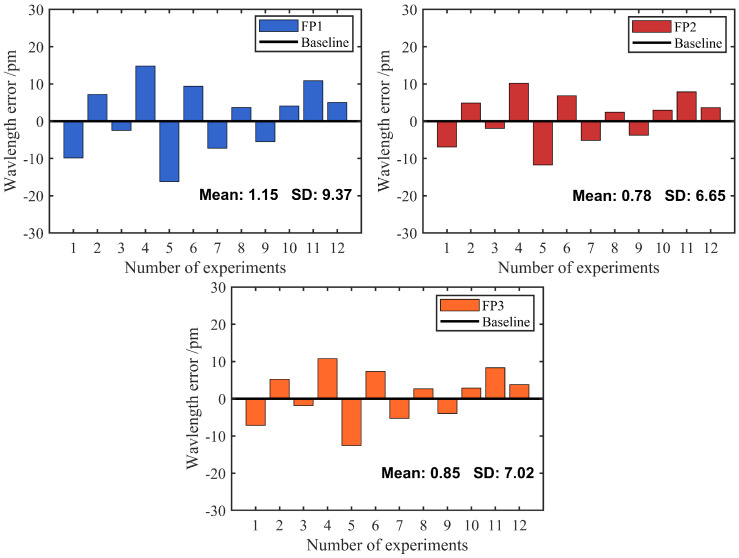
Error analysis results of twelve consecutive measurements of the FPIs.

To evaluate the stability of the proposed quasi-distributed sensing system, repeated experiments were conducted 12 times under the conditions of ${30\;}^{\circ }C$ ambient temperature and 35‰ seawater salinity using the three sensor units. Measurements were performed at 5 min intervals. The results are shown in Figure 8, where the bar chart intuitively displays the wavelength errors of the three sensor sets. It is evident that FP1 exhibits relatively large wavelength errors, with a mean value and standard deviation (SD) of 1.15 pm and 9.37 pm, respectively. The corresponding values for the other two FPIs are 0.78 pm and 6.65 pm, and 0.85 pm and 7.02 pm, respectively. The SD reflects the degree of dispersion of the sample values from their arithmetic mean. Generally, a smaller SD indicates a tighter data distribution, lower fluctuation, and more stable measurement performance. The mean and SD of all three FP sensors are on the picometer scale and in the single digits, demonstrating good stability of the fabricated sensors. Furthermore, to further assess the system stability, the limit of detection (LOD) was introduced as an indicator of sensitivity [33]. The LOD values of the three temperature sensors were −0.37 $\;\mathrm{pm}{/}^{\circ }C$, −0.40 $\;\mathrm{pm}{/}^{\circ }C$, and −0.51 $\;\mathrm{pm}{/}^{\circ }C$, respectively. These results indicate that the sensors maintain excellent detection stability within this multiplexed system.

## 4. Conclusions

This paper proposes a novel multi-point temperature-sensitivity-enhanced sensing method based on FMCW and the virtual Vernier effect, which has been experimentally validated. An FPI seawater temperature sensor with a specific optical path difference and misaligned splicing structure was fabricated. A DSP algorithm was employed for precise sensor localization and interference spectrum demodulation. The original interference spectrum of the FPI was subsequently modulated to generate an envelope line exhibiting the Vernier effect. Experimental results demonstrate that the proposed method achieves a long-distance sensing range of 82.3 m with a spatial resolution of 23.9 μm. Within the temperature range of 30–70 °C, the three FPIs exhibit high temperature sensitivities up to −280.67 pm/°C, corresponding to a 6.13-fold enhancement compared to a conventional single FP sensor. Furthermore, all three FP sensors demonstrated favorable stability in repeated tests. The proposed distributed sensitivity-enhanced sensing technique not only retains the high-precision localization capability of FMCW but also eliminates the need for physical reference interferometers to achieve Vernier-based enhancement, thereby fundamentally overcoming the limitations of traditional methods. While this study has focused on single-parameter temperature sensing at a fixed salinity, future work in this field should aim to achieve multi-parameter seawater sensing and eliminate the inherent cross-sensitivity.

## Figures and Tables

**Table 1:** Demodulation parameters and sensitization results of three groups of sensors.

Parameter	FP1	FP2	FP3	Env1	Env2	Env3
Range	${30\;}^{\circ }C$–${70\;}^{\circ }C$					
FSR (nm)	4.34	4.18	4.12	20.10	20.00	20.04
Modulation length (μm)	643	643	643	/	/	/
R^2^	0.99907	0.99875	0.99794	0.99998	0.99882	0.99882
Sensitivity ($\;\mathrm{pm}{/}^{\circ }C$)	−82.89	−54.67	−45.78	−275.56	−269.78	−280.67
Magnification *M*	3.32	4.93	6.13	/	/	/

## Data Availability

The raw data supporting the conclusions of this article will be made available by the authors on request.

## Abbreviations

FMCW	Frequency-modulated continuous wave
DSP	Digital signal processing
FPI	Fabry–Perot interferometer
SIS	Sensing interference spectrum
ARS	Artificial reference spectrum
EIS	Envelope interference spectrum

## References

[1] Roemmich D., Johnson G., Riser S., Davis R., Gilson J., Owens W.B., Garzoli S., Schmid C., Ignaszewski M. (2009). The Argo Program: Observing the Global Oceans with Profiling Floats. Oceanography.

[2] Abraham J.P., Baringer M., Bindoff N.L., Boyer T., Cheng L.J., Church J.A., Conroy J.L., Domingues C.M., Fasullo J.T., Gilson J. (2013). A Review of Global Ocean Temperature Observations: Implications for Ocean Heat Content Estimates and Climate Change. Rev. Geophys..

[3] Donlon C., Robinson I., Casey K.S., Vazquez-Cuervo J., Armstrong E., Arino O., Gentemann C., May D., LeBorgne P., Piollé J. (2007). The Global Ocean Data Assimilation Experiment High-Resolution Sea Surface Temperature Pilot Project. Bull. Am. Meteorol. Soc..

[4] Xue D., Zhang H., Wang S., Li H., Jiang J., Jia D., Liu T. (2023). High Sensitivity Composite F-P Cavity Fiber Optic Sensor Based on MEMS for Temperature and Salinity Measurement of Seawater. Opt. Express.

[5] Wu S.-C., Zheng H.-N., Li S.-Y., Wang Y., Tong R.-J. (2025). An SPR-MZ Interference-Based Fiber-Optic Sensor for Dual-Parameter Measurement of Seawater Temperature and Salinity. IEEE Sens. J..

[6] Ma Y., Han B., Liu R., Zhao J., Zhao Y. (2024). Dual-Channel Mach-Zehnder Interferometer Based on Femtosecond Laser Writing Waveguide for Seawater Temperature and Salinity Sensing. J. Light. Technol..

[7] Zhao Y., Zhao J., Zhao Q. (2020). High Sensitivity Seawater Temperature Sensor Based on No-Core Optical Fiber. Opt. Fiber Technol..

[8] Liang H., Wang J., Zhang L., Liu J., Wang S. (2022). Review of Optical Fiber Sensors for Temperature, Salinity, and Pressure Sensing and Measurement in Seawater. Sensors.

[9] Zhang S., Zhao Y., Peng Y., Zhao J.-C. (2024). High-Sensitivity Optical Fiber SPR Sensor with Cascaded Biconical Fiber and Hetero-Core Structure for Simultaneous Measurement of Seawater Salinity and Temperature. Opt. Laser Technol..

[10] Zhu Y., Sheng Q., Han M. (2020). Effect of Laser Polarization on Fiber Bragg Grating Fabry-Perot Interferometer for Ultrasound Detection. IEEE Photonics J..

[11] Xia Q., Zhang X., Zhang K., Wang H., Yuan L., Yuan T. (2025). Research on Fiber Bragg Grating Fabry-Perots Bending Sensor Based on Principal Component Analysis. Opt. Express.

[12] Liu Q., Yang D., Xing M., Yan C., Liu B. (2024). Simultaneous Measurement of Temperature and Pressure Sensing Technology Based on Double Cavity Matching in Batteries. Opt. Fiber Technol..

[13] Zhu T., Ke T., Rao Y., Chiang K.S. (2010). Fabry–Perot Optical Fiber Tip Sensor for High Temperature Measurement. Opt. Commun..

[14] Ding Z., Guo H., Liu K., Hua P., Zhang T., Li S., Liu J., Jiang J., Liu T. (2023). Advances in Distributed Optical Fiber Sensors Based on Optical Frequency-Domain Reflectometry: A Review. IEEE Sens. J..

[15] Chen T., Xu Z., Tu L., Wang L., Tang M. (2024). Improving OFDR Sensing Performance Based on SEFR in Both Reference and Measurement Stages. Opt. Lett..

[16] Liang C., Zhou X., Wang Y., Gao Y., Zhang H., Jin B., Bai Q. (2025). Sensing Distance Enhancement in OFDR Using Multi-Arms Interferometer Phase Noise Compensation Method. J. Light. Technol..

[17] Li P., Shang C., Tong Z., Wang H. (2025). Temperature Sensitivity Enhancement of OFDR Fibers Based on PDMS/Glycerol Films. Opt. Mater. (Amst.).

[18] Kong D., Chen C., Zhao X., Tao Y., Wan J., Wen Y., Zhang X., Yuan S., Liu X. (2024). Distributed Temperature Sensing on Silicon-on-Insulator Chip by Optical Frequency Domain Reflectometry. Opt. Express.

[19] Froggatt M.E., Gifford D.K., Kreger S., Wolfe M., Soller B.J. (2006). Characterization of Polarization-Maintaining Fiber Using High-Sensitivity Optical-Frequency-Domain Reflectometry. J. Light. Technol..

[20] Li J., Gan J., Zhang Z., Heng X., Yang C., Qian Q., Xu S., Yang Z. (2017). High Spatial Resolution Distributed Fiber Strain Sensor Based on Phase-OFDR. Opt. Express.

[21] Xu J.-J., Tang C.-S., Yang Y., Zeng Z.-J., Li L., Cheng Q., Zhang X.-Y., Shi B. (2024). Monitoring Soil Cracking Using OFDR-Based Distributed Temperature Sensing Framework. Geoderma.

[22] Sun Z., Xiao X., Zhao W., Ai K., Lv Y., Liu H., Sun Q., Yan Z. (2025). High-Precision Distributed Temperature Sensor Based on Phase-Shifted FBGs Interrogated by OFDR. J. Light. Technol..

[23] Zhu Z., Ba D., Liu L., Qiu L., Yang S., Dong Y. (2021). Temperature-Compensated Multi-Point Refractive Index Sensing Based on a Cascaded Fabry-Perot Cavity and FMCW Interferometry. Opt. Express.

[24] Zhu Z., Ba D., Liu L., Qiu L., Yang S., Dong Y. (2021). Multiplexing of Fabry-Pérot Sensor by Frequency Modulated Continuous Wave Interferometry for Quais-Distributed Sensing Application. J. Light. Technol..

[25] Feng Z., Tang Y., Cheng Y., Sima C., Yuan L. (2024). High-Spatial-Resolution Quasi-Distributed Fiber Sensing Technique Based on Cascaded FP Cavities and DSP Algorithm. J. Light. Technol..

[26] Shao L.-Y., Luo Y., Zhang Z., Zou X., Luo B., Pan W., Yan L. (2015). Sensitivity-Enhanced Temperature Sensor with Cascaded Fiber Optic Sagnac Interferometers Based on Vernier-Effect. Opt. Commun..

[27] Chen Y., Xu D., Xu K., Zhang N., Liu S., Zhao J., Luo Q., Snyman L.W., Swart J.W. (2019). Optoelectronic Properties Analysis of Silicon Light-Emitting Diode Monolithically Integrated in Standard CMOS IC*. Chin. Phys. B.

[28] Liu Y., Chen H., Chen Q., Li B., Li S. (2022). Experimental Study on Dual-Parameter Sensing Based on Cascaded Sagnac Interferometers With Two PANDA Fibers. J. Light. Technol..

[29] Yin G., Jiang R., Zhu T. (2022). In-Fiber Auxiliary Interferometer to Compensate Laser Nonlinear Tuning in Simplified OFDR. J. Light. Technol..

[30] Li Y., Yang Y., Mu X., Li Y., Zhang Y., Mo C., Xia H., Huang Z. (2024). Sensitivity-Enhanced Optical Fiber Sensor Based on the Vernier Effect for Detection of Ammonia in Water. Opt. Express.

[31] Fang X., Zhang W., Li J., Lin C., Chen Z., Zhang M., Huang S., Lu D., Wan M., Qiu X. (2021). Signal Processing Assisted Vernier Effect in a Single Interferometer for Sensitivity Magnification. Opt. Express.

[32] Zheng H., Lyu R., Zhao Y., Peng Y., Lin Z., Liu R. (2023). Research on the Distributed Measurement Method of Ocean Optical Fiber Sensor Based on Interferometer. IEEE/CAA J. Autom. Sin..

[33] Ozgur F.O., Çimen D., Denizli A., Bereli N. (2023). Surface Plasmon Resonance Based Sensor for Amaranth Detection With Molecularly Imprinted Nanoparticles. Photonic Sens..

